# Molecular mechanisms of metformin action: From metabolic effects to lifespan extension and healthspan promotion

**DOI:** 10.5937/jomb0-60849

**Published:** 2026-01-28

**Authors:** Slavica Vujović, Svetlana Perović, Milorad Vlaović, Staša Šćepanović, Aleksandra Filipović

**Affiliations:** 1 University of Montenegro, Faculty of Natural Sciences and Mathematics, Podgorica, Montenegro; 2 University of Montenegro, Faculty of Medicine, Podgorica, Montenegro + Private Health Centre Polyclinic Filipovic, Podgorica, Montenegro; 3 University of Montenegro, Faculty of Medicine, Podgorica, Montenegro; 4 University of Belgrade, University Clinic Centre of Serbia, Faculty of Medicine, Clinic of Gynaecology and Obstetrics, Belgrade; 5 General Hospital Celje, Resident of Internal Medicine, Celje, Slovenia; 6 Private Health Centre Polyclinic Filipovic, Podgorica, Montenegro

**Keywords:** anti-ageing, AMPK, longevity, metformin, mitochondria, mTOR, AM PK, dugovečnost, metformin, mitohondrije, mTOR, starenje

## Abstract

**Background:**

Metformin, a biguanide primarily used for the treatment of type 2 diabetes mellitus, has attracted significant attention for its potential anti-ageing effects. As ageing becomes the primary risk factor for chronic diseases, interventions targeting fundamental ageing processes are gaining traction in biomedical research.

**Methods:**

Accumulating evidence suggests that metformin exerts geroprotective effects through multiple interconnected pathways. These include activation of AMP-activated protein kinase (AMPK), inhibition of the mechanistic target of rapamycin (mTOR), attenuation of oxidative stress, modulation of mitochondrial biogenesis, and reduction of low-grade systemic inflammation. Together, these actions address key hallmarks of ageing such as cellular senescence, dysregulated nutrient sensing, and altered proteostasis.

**Results:**

Animal studies have consistently shown that metformin extends both lifespan and healthspan. In humans, retrospective epidemiological data indicate reduced incidence of cancer, cardiovascular disease, and cognitive decline among metformin users. The TAME (Targeting Ageing with Metformin) trial represents the first large-scale attempt to assess ageing-related outcomes in a non-diabetic population formally. Despite promising data, uncertainties remain regarding optimal dosing, long-term safety, and applicability in healthy ageing populations. Furthermore, individual variability in response to metformin suggests the need for precision medicine approaches.

**Conclusions:**

Metformin stands at the intersection of metabolic regulation and ageing biology. While not a panacea, its favourable safety profile and multi-targeted actions make it a leading candidate for repurposing as an anti-ageing therapy. Continued clinical validation is essential to translate these insights into practice.

## Abstract (sr)

**Uvod:**

Metformin, bigvanidni lek koji se prvenstveno koristi u terapiji dijabetesa tipa 2, u poslednje vreme privlači sve veću pažnju zbog svog potencijalnog geroprotektivnog delovanja. S obzirom na to da starenje predstavlja primarni faktor rizika za razvoj hroničnih bolesti, intervencije usmerene na temeljne biološke procese starenja postaju sve značajnije u okviru savremenih biomedicinskih istraživanja.

**Metode:**

Sve veći broj istraživanja ukazuje na to da metformin ostvaruje geroprotektivne efekte putem više međusobno povezanih molekularnih puteva. Među njima se izdvajaju: aktivacija AMP-aktivirane proteinske kinaze (AMPK), inhibicija mehanističkog cilja rapamicina (mTOR), smanjenje oksidativnog stresa, modulacija biogeneze mitohondrija i redukcija hronične upale niskog intenziteta. Ovi mehanizmi zajednički deluju na ključne karakteristike starenja, uključujući celijsku senescenciju, poremećenu nutritivnu signalizaciju i disbalans u homeostazi proteina.

**Rezultati:**

Studije na animalnim modelima dosledno pokazuju da metformin produžava životni vek i poboljšava zdravlje u starosti. Kod ljudi, retrospektivne epidemiološke analize ukazuju na smanjen rizik od razvoja maligniteta, kardiovaskularnih bolesti i kognitivnog propadanja kod korisnika metformina. Studija TAME (Targeting Ageing with Metformin) predstavlja prvi sveobuhvatni pokušaj da se proceni uticaj metformina na ishode povezane sa starenjem u populaciji koja nije obolela od dijabetesa. Uprkos ohrabrujućim nalazima, i dalje postoje nedoumice u vezi sa optimalnim režimom doziranja, dugoročnom bezbednošću primene, kao i primenljivošću metformina u opštoj, zdravoj starijoj populaciji. Dodatno, interindividualne razlike u odgovoru na terapiju ukazuju na potrebu za razvojem pristupa zasnovanih na principima personalizovane medicine.

**Zaključak:**

Metformin se nalazi na raskršću metaboličke regulacije i biologije starenja. Iako nije univerzalni lek, njegov povoljan bezbednosni profil i višesmerni molekularni mehanizmi čine ga jednim od najperspektivnijih kandidata za prenamenu u svrhe usporavanja starenja. Potrebna su dodatna klinička ispitivanja kako bi se ovi nalazi potvrdili i implementirali u kliničku praksu.

## Introduction

Ageing is characterised by a gradual decline in the function of physiological tissues and is linked to numerous chronic diseases that affect humans. As individuals grow older, their likelihood of developing various chronic illnesses increases, including cancer, osteoporosis, cardiovascular diseases, and neurodegenerative disorders.

The widely accepted biological definition of ageing involves »a decline in the ability to regenerate damaged tissue« and the progressive deterioration of homeostatic processes over time. This ultimately causes functional decline, a higher risk of many diseases, and can also lead to a fatal outcome [1]. These chronic diseases and age-related physiological changes are the main causes of illness and death in humans. For example, sarcopenia and osteoporosis result in significant loss of muscle and bone mass due to dominant catabolic activity, leading to more frequent injuries in older patients and increased severity of clinical consequences.

Systemic inflammation that increases with age (»inflammaging«) plays a key role in the onset and progression of many chronic conditions. It predisposes older individuals to arthritis, autoimmune disorders, and other degenerative diseases by inducing cellular senescence, immunosenescence, and organ dysfunction [2] [3]. These pathological processes are commonly referred to as age-related diseases (ARDs) in the literature.

Treating each of these diseases separately is often impractical, even when feasible, and it is essential to consider the overall financial burden to attain meaningful results. Since the ageing process itself is one of the most significant risk factors for chronic diseases, slowing or delaying the biological mechanisms of ageing could bring substantial medical and socioeconomic advantages [4].

There is substantial evidence supporting that ageing is linked to both genetic and epigenetic alterations. Traditionally, biomolecular models of ageing have been divided into two paradigms: the first proposes that ageing is genetically programmed through developmental processes, such as cell senescence, neuroendocrine changes, and immunological modifications, while the second attributes ageing to the stochastic accumulation of cellular damage, particularly somatic mutations and oxidative stress (OS). Recent research has increasingly emphasised that these mechanisms are not mutually exclusive, but rather act synergistically in driving the ageing process [5] [6].

Moreover, chronic low-grade inflammation and oxidative stress are interconnected hallmarks of ageing, contributing to mitochondrial dysfunction, genomic instability, and tissue degeneration. The accumulation of numerous molecular and cellular changes that occur during ageing progressively reduces the organism's ability to cope with environmental and metabolic stressors. This gradual build-up of damage increases vulnerability to functional decline, disease, and mortality - first at the cellular level and subsequently at the systemic level [7] [8].

This natural phase of the human life cycle exerts enormous pressure on medical systems, human productivity, and the global economy. According to the WHO, between 2020 and 2050, the global population aged 60 years and older is expected to double to 2.1 billion, while the number of people aged 80 and older will triple to 426 million [9]. Such demographic changes will inevitably increase healthcare costs and strain public health infrastructure. Tang et al. [10] demonstrated that between 2000 and 2019, each 1% increase in healthcare costs associated with an ageing population corresponded to a 0.083% decrease in the GDP growth rate.

ARDs such as cardiovascular diseases, neurodegenerative disorders, and cancer significantly affect human independence, overall well-being, and morbidity on the individual level. However, on a population scale, these diseases transcend biology, shaping productivity, healthcare systems, lifestyles, and social dynamics [11]. Therefore, a deeper understanding of the molecular mechanisms underlying ageing is essential for developing interventions that promote healthy longevity and reduce the burden of age-related diseases.

## Materials and methods

### Literature search strategy

The conduct and reporting of the current systematic review followed the PRISMA (Preferred Reporting Items for Systematic Reviews and MetaAnalyses) guideline. This review aimed to improve the transparency and quality of systematic reviews and included three main stages, namely: identification, screening and inclusion. A comprehensive literature search was conducted using PubMed, Scopus, and Web of Science databases to identify relevant studies on the molecular mechanisms underlying metformin's anticancer and anti-ageing effects. Although some landmark studies published earlier were considered, the primary focus was on literature from the last ten years (2014-2024) to ensure up-to-date scientific coverage. The following keywords and their combinations were used: metformin, anticancer, anti-ageing, AMPK, mTOR, oxidative stress, cellular senescence, mitochondria, and metabolic reprogramming.

### Inclusion and exclusion criteria

Included studies were: (1) peer-reviewed original research articles or review papers published in English; (2) studies addressing the molecular or cellular mechanisms of metformin in metabolism and/or ageing contexts; (3) clinical models. Articles were excluded if they were editorials, conference abstracts, non-English publications, or lacked a molecular focus relevant to the topic.

### Study selection process

After initial screening of titles and abstracts for relevance, full-text articles were assessed. Studies were selected based on their scientific quality, novelty, and direct relevance to metformin's biological mechanisms in ageing and metabolism. Priority was given to high-impact publications and recent advances in the field. Duplicates and irrelevant studies were removed during the selection process.

### Data extraction and synthesis

Selected articles were systematically analysed to extract information about key molecular pathways influenced by metformin, including AMPK activation, mTOR inhibition, regulation of oxidative stress, mitochondrial function, and cellular senescence. Findings were categorised thematically to provide a coherent narrative on the overlap and divergence between metformin's metabolic effects and anti-ageing actions.

## Results and discussion

### Role of metabolism in ageing, and hallmarks of ageing

Nowadays, there is an increasing amount of research showing the strong connection between ageing and diabetes, particularly type 2 diabetes mellitus (T2DM). It is believed that ageing and diabetes lead to similar organ dysfunctions, triggered by numerous molecular mechanisms, one of which is cellular ageing. The increase in senescent cells in various tissues occurs with age and the progression of diabetes. These cells are directly involved in the creation of insulin resistance. Additionally, the biological age (BA) appears to correlate with cellular changes, which are more pronounced in diabetic (type 1) and T2DM patients, increasing their estimated BA by 16 and 12 years, respectively, compared to non-diabetic controls [12]. It is for this very reason that treatment strategies targeting both ageing and diabetes/prediabetes/insulin resistance (IR), such as metformin, are a promising avenue in anti-ageing research and medicine.

Dysregulated metabolism is, in fact, one of the many hallmarks of ageing, characterised by typical metabolic syndrome/insulin resistance features attributed to ageing. Recent research implies age-related metabolic changes may play a more critical role than they were previously considered to, while also strongly suggesting we may no longer be able to blame weight gain in middle age on the so-called »slowed metabolism«. The basal metabolism rate seems to remain pretty stable between 20 and 60 years of age, whereas older individuals show reduced energy expenditure [13] [14].

Hallmarks of ageing are cellular and molecular processes linked to degenerative physiological and anatomical changes that occur with age. These processes can be experimentally enhanced or suppressed through targeted therapies, thereby shortening or extending both lifespan and healthspan. Depending on the authors and school of thought, there are anywhere between 8 and 12 hallmarks of ageing, typically including: genomic instability, telomere attrition, epigenetic alterations, loss of proteostasis, deregulated nutrient-sensing, mitochondrial dysfunction, cellular senescence and stem cell exhaustion. Some authors add chronic inflammation, disabled macroautophagy, altered intercellular communication, oxidative species signalling imbalance (oxidative stress) and even dysbiosis to these. They are sometimes further classified and their taxonomy expanded to illustrate how some of them cluster together mechanistically. For example, they can be divided into 3 major categories: primary, integrative and antagonistic. This, however, does not mean they function separately and independently to cause ageing. On the contrary, experimental data suggest that affecting even one of the hallmarks will usually impact several others as well. So in that sense, these processes are interdependent. Some age-related cellular/molecular changes almost always occur in tandem, supporting one another in a vicious cycle, such as oxidative stress and inflammation [11] [15] [16] [17].

### Oxidative stress, mitochondrial instability, inflammation and ageing

The free radical theory of ageing is one of the most reliable models explaining some crucial mechanisms of the ageing process in mammals. According to this theory, an excess of free radicals in biological systems, such as reactive oxygen and reactive nitrogen species (ROS and RNS, respectively), is associated with many diseases linked to ageing. Scientists have focused their attention on the possibility of increased ROS levels along with changes in redox balance, which ultimately may lead to chronic inflammation. Numerous scientists have proposed the »Ageing and Molecular Inflammation Hypothesis« as a potential link between biological ageing and pathological conditions associated with ageing. ROS levels and redox imbalance trigger intracellular signalling cascades that can potentially lead to chronic inflammation, contributing to the ageing process and the manifestations of various diseases associated with ageing. To a certain point, ROS/RNS signalling may indeed elicit a homeostasis/restoration response, beneficial in extending age and healthspan, but passing this point, their effects become deleterious to healthspan instead [18] [19].

Of particular importance for the ageing process are the mitochondrial reactive oxygen species (mtROS). These can be produced in at least 11 specific mitochondrial locations. While inherently not harmful and even acting as signalling molecules triggering important physiological processes, they shift the redox balance to increased ROS and a decreased antioxidant state in excess. Usually, the increased production of mtROS is due to a stressor, such as thermal stress, and its removal will result in the process resolving itself. In the case of »old« mitochondria, however, the increase in mtROS production is a continuous process independent of stress induction. mtROS production, like many other processes, is therefore clearly dysregulated during ageing, interfering with the detection of ROS signals from mitochondria and elsewhere, which is a more problematic mechanism in terms of ageing (it contributes to both mitochondrial disfunction and dysregulated intracellular signaling) than mere oxidation of biological molecules, as some of those molecules may be salvaged by means of »recycling«. Of course, the damage to cellular lipids, proteins, and DNA (to name a few biomolecules) is far from negligible and clearly contributes to the ageing phenotype. Elevated ROS levels can cause oxidative DNA damage that results in mutagenesis (therefore, crosslinking with the genetic instability hallmark). Additionally, the same oxidative DNA damage can be induced in bystander cell populations (cell populations neighbouring or sharing media with damaged or stressed cells) [20] [21] [22].

The free radical theory of ageing, proposed in 1955 by Harman, because mitochondria are major sources of ROS and RNS, but also a target for them, resulting in dysfunction typical for the ageing phenotype, has been revised by the same author as the mitochondrial free radical theory of ageing (MFRTA) [23].

A shift in the redox balance and ROS and RNS excess, by very means of causing oxidative damage to essential biomolecules, triggering and supporting low-grade systemic inflammation (LGSI). Numerous studies prove these two processes (oxidative stress and inflammation) to be interdependent and often causative of one another in a vicious cycle. Inflammatory cells secrete various reactive species at the inflammation site, leading to exaggerated oxidative stress. Similarly, several ROS/RNS species can initiate an intracellular signalling cascade that will ultimately upregulate the activity of the proinflammatory genes. Cytokines and nuclear factor kappa beta (NF-kB) are some of the most common mediators between oxidative stress signalling and inflammatory pathways. The NOD-like receptors (NLR) and Toll-like receptors (TLR) are also of great importance here [24] [25].

### Aberrant intracellular signalling of AMPK, IGF1 and mTOR

Dysregulation in signalling cascades within a single cell, alongside extracellular communications (hormones, neurotransmitters), has been widely implicated as an important factor in the ageing process, with some molecules being of particular interest (e.g., AMPK, Insulin, IGF, and mTOR) as potential therapeutic targets.

Studies in mice have demonstrated that age-related reduction in AMPK enzyme activation may be one of the essential causes of mitochondrial dysfunction and dysregulated lipid metabolism in mammalian cells, and its signalling clearly declines with age. Such a decline also activates innate immunity defensive response, triggering LGSI and metabolic disorders [26] [27].

AMPK activation is beneficial for cellular homeostasis and even ageing prevention. Many studies have suggested that AMPK plays a significant role in preventing ageing. AMPK signalling activates autophagy. The most commonly described mechanism involves AMPK suppressing the mTORC1 pathway. Several pharmacological AMPK activators, such as metformin, are characterised as having the potential to treat metabolic, neurodegenerative, and many other diseases related to ageing. It has been shown that NAD+ decreases with ageing, but it is still unclear why this happens. Interestingly, AMPK activation increases NAD+ levels and activates SIRT1, which possesses Nicotinamide Phosphoribosyltransferase (NAMPT), the key enzyme involved in the NAD+ synthesis pathway [28].

Numerous studies have shown that AMPK plays a crucial role in regulating longevity. Studies have confirmed several AMPK mechanisms linked to lifespan extension, including the inhibition of CRTC-1/CREB, NFkB, and mTORC1, and the activation of SRT1, NRF2/SKN1, FOXO1/DAF-16, and ULK1, which induce antioxidant defence against inflammation and autophagy [27] [29].

Insulin and IGF-1 signalling are crucial for growth. Insulin (or insulin-like growth factor, IGF-1) is responsible for this process. IGF-1 insulin receptor (InR) activates a cascade of kinases and phosphatases, including PI3K, PTEN, PDK, SGK, and AKT, which leads to the inhibition of the transcription factor FOXO. In worms, flies, and mice, it has been shown that disrupting insulin-IGF-1 signalling extends lifespan. In these organisms, FOXO regulates the transcription of many genes involved in stress response and resistance to bacteria, which contribute to the longevity of Caenorhabditis elegans. An excess of FOXO is enough to extend the lifespan of fruit flies. In mammals, the FOXO proteins, particularly FOXO3, are linked to lifespan extension. Their upregulation is associated with stress resistance, balancing metabolism, and cell cycle arrest, contributing to healthy ageing and longevity, and is expected to have similar effects in humans too [30] [31] [32]. Conversely, overly active IGF-1 pathway signaling is inversely correlated to human lifespan and healthspan, according to some cohort studies, such as one conducted by Milman et al. [33]. Also, a large-scale study involving nearly 450,000 UK biobank participants demonstrated that older adults with higher IGF-1 levels had a greater risk of mortality and ARD, suggesting that lower IGF-1 levels conferred better survival [34]. Diminished IGF-1 receptor expression has even been shown to extend lifespan in model organisms and in humans [35].

Another critical signalling pathway that affects ageing is mTOR (mechanistic target of rapamycin). mTOR is a member of the kinase family associated with PI3K. TOR exists in two complexes, mTORC1 and mTORC2. mTORCI is inhibited by rapamycin, while mTORC2 is not affected by rapamycin and controls cell shape. Amino acids and insulin-IGF-1 activate mTORCI through the kinase AKT, which represents the main convergence point between insulin-IGF-1 and mTOR. mTORC1 activates more anabolic processes, including ribosome biogenesis, nutrient import, and inhibition of the catabolic process of autophagy [30].

It has been shown that mTORC1 signalling affects ageing in many organisms. Reduced mTOR activity extends lifespan in yeast, mice, fruit flies, and worms. Two processes regulated by mTOR are associated with a longer lifespan. The first is protein synthesis, regulated by the ribosomal protein S6 kinase (S6K). Inhibition of S6K reduces protein synthesis and has been shown to extend lifespan. The second process is autophagy. In general, mTOR has been implicated in many age-related processes, including cellular senescence, immune responses, stem cell activity regulation, autophagy, mitochondrial function, and protein homeostasis (proteostasis). Compounds that limit or reduce their activation, such as rapamycin, metformin, berberine and others, have been shown to extend lifespan and improve function in the model organisms [30] [36] [37].

### Cellular ageing/senescence

Cellular ageing (also known as senescence) is the cessation of the cell cycle that occurs in differentiated cells, limiting their proliferative lifespan. Senescent cells that emerge because of this process can accumulate with age, leading to pathological changes associated with ageing. It is an irreversible form of long-term cell-cycle arrest caused by excessive exogenous stress or damage, or by damage originating from inside the cell itself. This arrest is sometimes a desired physiological response, for example, in preventing the division and proliferation of transformed cells [38] [39]. So, besides being a mechanism for tumour suppression, it also inhibits key mediators in the ageing process, such as p53 [40].

Cellular ROS production is a common sign of apoptosis and senescence. Many compounds have been tested for their activity to remove/kill senescent cells. Recent studies have shown that metformin weakens cellular apoptosis and induces senescence in nucleus pulposus cells when used with tert-butyl hydroperoxide, suggesting that metformin can be considered a senolytic drug [41]. However, senolytics often act on other anti-ageing target molecules and pathways (ex., Quercetin in combination with Dasatinib acts as a senolytic, but also targets the mTORC1), so it is hard to attribute their life-extension effects to simply the removal of senescent cells. The real goal would not be merely to destroy these cells, but rather to prevent the geroconversion of healthy cells into senescent ones. After all, selective destruction of senescent cells is very hard to accomplish [42].

Senescent cells are »problematic« in terms of ageing because their nuclei undergo epigenetic changes (»reprogramming«) that upregulate the Senescence-Associated Secretory Phenotype (SASP). This causes them to release extracellular modulators such as cytokines, chemokines, proteases, and growth factors into the surrounding tissue. These molecules act dynamically; they can either suppress neoplastic changes or support them. When it comes to ageing, an overactive SASP is generally linked in the literature with a shorter lifespan and poorer quality due to an increase in LGSI, among other factors [8] [43].

It seems however, that when it comes to cellular ageing as a target for the anti-ageing intervention, the approach cannot be simplified to merely eliminating the senescent cells, but also selective modulation of their activity (SASP inhibition), or targeting the chimeric antigen receptors (CAR) on their surface (immunotherapies and »senovacines«) and so forth [44].

Metformin, a biguanide, may offer a solution. This drug is reported to combat ageing-related disorders and improve overall health. It is the first drug tested for effects targeting ageing in a large clinical trial - TAME (Targeting Ageing with Metformin) [45].

### What is metformin, and how does it work

Metformin, a biguanide, is the most commonly used medication for type 2 diabetes (T2DM), as a first-line treatment, especially in cases of patients where the diet and exercise have failed to regulate the glucose levels adequately, particularly in the obese and overweight patients. It targets fat tissue, liver and muscles (among others), and typically manages to lower blood glucose without the weight gain or risk of overt hypoglycemia. It is currently also being used in the treatment of polycystic ovary syndrome (PCOS) and non-alcoholic fatty liver disease (NAFLD). It is being considered for repurposing in the treatment of neurological conditions, as the evidence supporting such use is both already rich and continually emerging [22] [46].

It is derived from a natural compound synthesised by the plant Galega officinalis, which was traditionally used as an herbal remedy to alleviate symptoms now associated with diabetes. It has a molecular mass of 129 daltons [47]. This plant contains guanidine, a compound shown to have hypoglycemic properties [48]. Metformin, with its delayed-release formulation, works in the lower gastrointestinal tract and reduces glucose concentrations in patients with renal disease [49].

Its antihyperglycemic effect is mediated by increasing insulin sensitivity in the liver and glucose absorption in muscle cells. Mitochondria play a significant role in metformin's mechanism. The main function of metformin is ATP production via oxidative phosphorylation [50].

In addition to its primary function of lowering glucose levels, metformin offers other benefits, such as improving lipid profiles, reducing markers of inflammation, lowering the risk of cardiovascular disease, and potentially having an anti-ageing effect, which is considered one of its most significant benefits [51].

Literature also suggests that metformin suppresses the inflammatory response by inhibiting the nuclear factor kB (NFkB) through AMPK-dependent and independent pathways [52].

While numerous mechanisms underlying metformin's effects are not yet fully understood, one of the most accepted mechanisms is the activation of AMP-activated protein kinase (AMPK). Recent studies suggest other mechanisms independent of AMPK that are important for metformin's action [53]. In T2DM, metformin's action in the liver inhibits gluconeogenesis, thereby lowering blood glucose levels and reducing elevated insulin levels, which are characteristic of the condition [54].

### Metformin: A metabolism stabilising and antiageing drug

In recent years, there has been an increasing number of studies focusing on the anti-ageing effects of metformin. It is believed that metformin plays a significant role in, and may lead to, extending life and preventing ageing, as well as various age-related diseases. This biguanide is a drug that effectively combats ageing-related disorders and improves overall health. It is the first drug tested for effects targeting ageing in a large clinical trial - TAME (Targeting Ageing with Metformin) [45].

Considering that several pathways that play a crucial role in longevity and ageing, such as IGF-1 and mTOR, are targets for metformin's activity, drugs like metformin (and rapamycin and similar drugs) are being researched as potential anti-ageing agents. Caloric restriction is also an important factor in the longevity pathway and life extension, and metformin was found to elicit a similar transcriptional response (in experimental model animals) as caloric restriction. Some even consider it a caloric restriction mimetic [55] [56] [57] [58].

**Figure 1:**
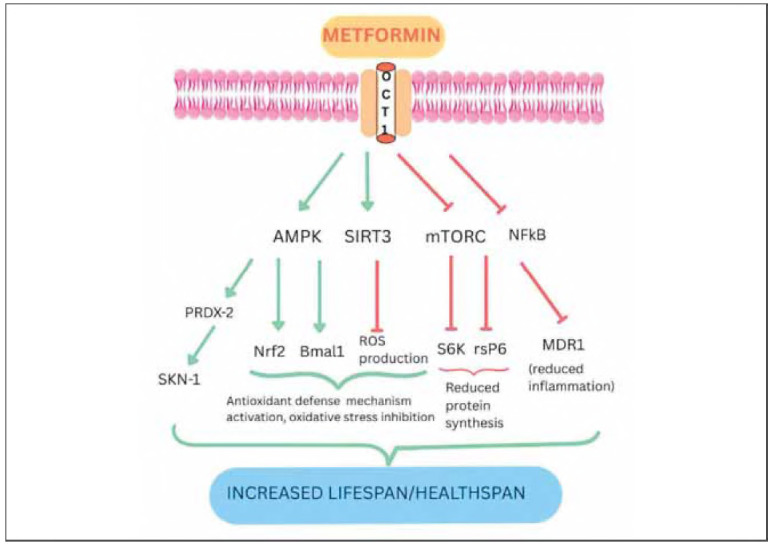
Example of some of the metformin's activation (green arrow) and inhibition pathways (red line) interplay in enabling its anti-ageing effects. PRDX-2 - peroxiredoxins coding gene, SKN-1 C. elegans analogue to human NRF2, Bmal1 - Basic helix-loop-helix ARNT-like protein 1, a circadian rhythm regulating protein, MDR1 - multidrug resistance gene, coding for the P-glycoprotein.

When it comes to the ageing process, metformin leads to a decrease in insulin levels, affects cytokine receptors, insulin, IGF-1, and adiponectin (a hormone responsible for regulating glucose and insulin levels and increasing fatty acid oxidation), inhibits mTOR pathway, and inhibits complexes in mitochondria that are important for electron transfer, reducing the endogenous production of ROS, and activates AMPK (Figure 1). It also inhibits the mTOR indirectly by exerting one of its metabolic effects, through alteration of passive transport across the nuclear pore complex. This prevents RagC from passing through the nucleus and gaining GDP-bound activity necessary for mTORCI activation. Through several of these mechanisms, metformin impacts oxidative stress by eliminating senescent cells (although the mechanism for this removal is still not fully explained) [59] [60].

Metformin seems to have a profound effect on oxidative stress - it directly removes ROS or indirectly modulates intercellular ROS production without the involvement of superoxide anion radicals. ROS are known to damage DNA molecules, and metformin helps repair this DNA damage, which is critical for maintaining normal metabolism and managing the ageing process and age-related conditions [61].

Continuing, we shall briefly discuss some of metformin's general mechanisms of action and how they may connect to its proposed anti-ageing activity. We will then go into more details on mechanisms recognised in the literature to enable its lifespan extension benefits, whether dependent on its metabolic effects or not.

### Inhibition of the mitochondrial respiratory chain

AMPK is a heterotrimeric serine/threonine protein kinase. It can be activated and phosphorylated at Thr172 by calcium/calmodulin-dependent kinases and Ca^2+^, but there is also an AMP-independent activation method [62]. By partially suppressing the electron transport chain's complex I in mitochondria, the ratio of AMP to ATP changes in such a manner (in favour of the AMP) that triggers the activation. This, in turn, restores the energy balance and helps mitochondrial health and function [63]. In this way, metformin use favorably affects mitochondrial dysfunction (which happens to increase with age naturally), one of the major hallmarks of ageing [64], as well as oxidative stress that can be a result of increased reactive oxidative species (ROS) »leakage« from the respiratory chain that in itself contributes to ageing [22] [65].

### AMP: AMPK-dependent and independent mechanisms

Metformin does not stimulate insulin secretion from pancreatic -cells, so it is a medication that does not cause hypoglycemia. Metformin affects cellular respiration by inhibiting mitochondrial respiratory chain complex I, leading to a decrease in intracellular ATP and an increase in AMP This process results in the stimulation of AMP-activated protein kinase (AMPK). AMPK is activated even with a minimal ATP deficit. This leads to the downregulation of all metabolic pathways that consume energy, resulting in the intensified translocation of the glucose transporter GLUT to the cell membrane, which enhances glucose uptake into the cell [66]. By mimicking caloric restriction and stimulating AMPK activity, metformin already contributes to extending lifespan and healthspan.

Another mechanism of respiratory chain inhibition is independent of AMPK activation. This mechanism is attributed to the reduction in blood glucose and insulin levels [67]. Some of these mechanisms may also explain the anticancer effects of metformin. The drug can inhibit DNA damage by preventing ROS formation via complex I. Metformin can also inhibit mTORCI activation in the absence of AMPK. Some studies provide evidence that the anti-neoplastic effect of metformin is mediated by the inhibition of cyclin D1, independent of AMPK, which plays a crucial role in regulating the cell cycle [50]. By reducing mitochondrial ROS formation and helping regulate the cell cycle in such an AMPK-independent way, metformin also reduces the chances of ARD occurrence.

### Autophagy induction

Autophagy stimulation/induction is another fundamental mechanism of action of metformin, one associated with AMPK activation. Autophagy refers to the process of degradation and removal of damaged organelles and proteins, including microautophagy. Autophagy is initiated by phosphatidylinositol-3 kinase of class III (PI-3K). It is a two-phase catabolic process: the formation of the autophagosome (1st phase) and the fusion of the autophagosome with lysosomes, resulting in the formation of an autophagolysosome (2nd phase) [68].

Autophagy can function selectively, meaning that any cytoplasmic content may become targeted for catabolic degradation, as is the case when the supply of nutrients is limited. Additionally, there are highly selective forms of autophagy that specifically target damaged organelles. A good example of this is mitophagy, which results in the »cleaning« of damaged mitochondria. Mitochondria are involved in essential and even lethal cell functions, and mitochondrial dysfunction is a critical determinant in the lifespan of species [69]. It is important to note that autophagy participates in a wide range of physiological and pathophysiological processes, such as growth, development, metabolism, inflammation, neurodegenerative diseases, cancer, and many cardiovascular diseases [68].

Metformin can activate or inhibit autophagy primarily by targeting the mTORCI and AMPK. Some of the mechanisms through which metformin affects autophagy are AMPK-independent. For example, the literature consistently reports that metformin can downregulate the STAT3 pathways and inhibit the expression of STAT3 mRNA and protein, resulting in metformin-induced autophagy. Also, metformin can induce autophagy through the AMPK-independent SIRT1 pathway, or conversely, inhibit it via the AMPK/nuclear factor kappa-B (NF-kB) pathway, the Hedgehog pathway (which is AMPK-independent), or by affecting several microRNAs [70]. Through the STAT3 pathway, and independently of it, metformin may also ameliorate inflammation (in the T cells at least) due to the Th17 inflammaging profile by increasing autophagy and improving mitochondrial bioenergetics, thus promising as a life-extension modification [71]. Additionally, through its autophagy-inducing virtue, metformin use tackles some of the autophagy related hallmarks of ageing via several mechanisms: it inhibits senescence (for example in the nucleus pulposus cell and periodontal ligament cell's), enhances mitophagy by upregulating mitophagy-related genes and enhances the LC3-II-mediated autophagy in dopaminergic neurons, helping prevent some neurodegenerative conditions, such as Parkinson's disease [72].

### Effects on the liver and lipid metabolism regulation

Studies in humans indicate that metformin reduces glucose production in the liver with minimal impact on peripheral glucose uptake mediated by insulin [68]. Metformin may also play a role in preventing liver injury induced by fructose. Mice were divided into groups treated with fructose. After 10 days of metformin treatment, the outcome showed that fructose resulted in hepatic steatosis, insulin resistance, and decreased insulin sensitivity, in combination with higher mRNA levels associated with de novo lipogenesis. In contrast, metformin reduced de novo lipogenesis, increased beta-oxidation, and also provided excellent defence against excessive ROS generation [73].

In line with these findings, by reducing the risk of liver steatosis and injury, metformin may also enhance lipid metabolism. Recent studies have shown that metformin lowers circulating lipids, thereby decreasing triglycerides in plasma through a selective increase in VLDL-triglyceride uptake and the oxidation of fatty acids in brown adipose tissue [74]. Research by Boyle et al. [75] demonstrated that metformin activates AMPK in human tissues in vivo, with this relationship clearly shown in adipose tissue. Other studies have indicated that adiponectin stimulates AMPK; however, this research found that AMPK activity in individuals treated with metformin was not associated with significant changes in plasma adiponectin levels, indicating that AMPK activity is independent of adiponectin [75].

Metformin shields the liver from oxidative damage and hepatotoxicity caused by I/R. This is partly achieved by lowering ROS production in mitochondria, which reduces mitochondrial damage and lessens necrotic cell death and inflammation. A study involving 208 Indian diabetic patients demonstrated that metformin restores the antioxidant response to oxidative stress in plasma. It is believed that metformin treatment effectively safeguards the liver from acute oxidative injuries, particularly when the liver is under stress [76].

Metformin is hepatoprotective in a model of insulin-resistant and leptin-deficient mice with NAFLD. The protective effects of metformin against oxidative stress and stress-induced apoptosis were investigated. In this study, Rosa et al. [77] exposed primary hepatocytes to a compound that generates oxidative stress, both with and without metformin. It was found that metformin did not induce necrosis in primary hepatocytes and protected them from apoptosis induced by oxidative stress. These results clarify the protective mechanism of metformin and suggest that metformin may be investigated as a new therapeutic agent for treating oxidative stress-related liver diseases [77].

Considering that genome wide association studies have found several lipid related variants to be associated with human ageing, as well as specific sphingolipid and phospholipid blood profiles, and that targeted interventions in lipid metabolism will extend lifespan and improve parameters of healthspan (at least in model organisms) [78], the aforementioned metabolic effects of metformin on liver and lipid metabolismare warranting research of its anti-ageing use through the exact mechanisms. Additionally, hepatic steatosis and NAFLD, with or without an increased risk of dyslipidemia and T2DM, are more common in the 60+ age group and are considered types of ARD [79]. Metformin has proven to be at least somewhat protective against these conditions.

## Metformin's effects on lifespan, healthspan and ARD outcome

Considering all the evidence, the claims that metformin may extend lifespan in humans remain highly controversial. While the preclinical (animal models) provide a plethora of positive results, these cannot be extrapolated to the human population.

However, clinical trials such as MILES (Metformin in Longevity Study) and TAME (Targeting Ageing with Metformin) are offering promising preliminary results, particularly regarding the transcriptome, as metformin appears to induce anti-age transcriptional changes, at least among patients with conditions such as T2DM. This, though, still leaves questions about its effects on healthy individuals and whether the risk of treatment in this subset of the population is worth it [80] [81]. Metformin is the first drug tested for effects targeting ageing in a large clinical trial - TAME [45].

**Figure 2:**
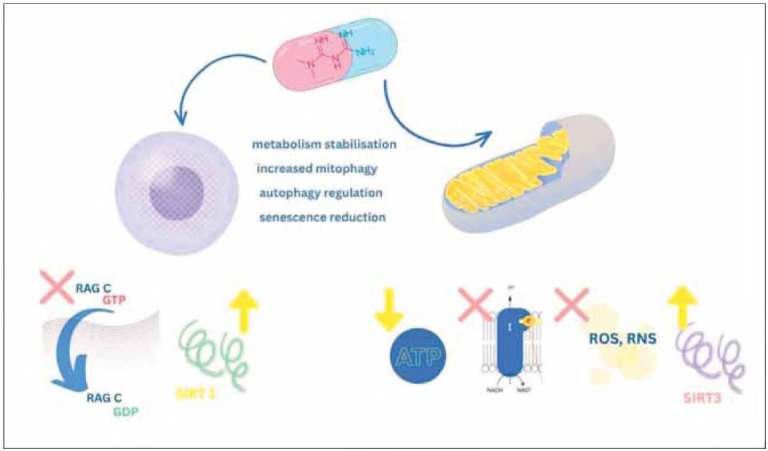
Nuclear and mitochondrial metabolic effects of metformin, and the processes that result from them, which contribute to its life/healthspan-increasing effects.

**Figure 3:**
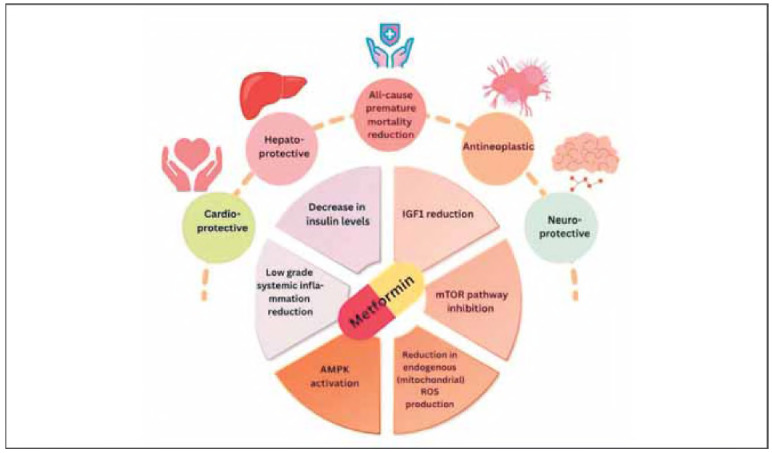
Metformin's physiological activities related to healthspan and the underlying molecular mechanisms unifying and enabling these effects.

Metformin's effects on healthspan might actually be far more valuable than its effects on mere life extension. Via its ability to reduce premature all-cause mortality associated with various ARD, including diabetes, cardiovascular disease, cognitive decline/ dementia and cancer, metformin can extend and improve the period of life spent in good health, providing cardioprotective, neuroprotective and anti-neoplastic benefits [82] (Figure 2). Table 1 Figure 3

**Table 1:** Examples of metformin's ARD-protective mechanisms on a molecular and physiological level in selected organs/organ systems [45].

Orgman or organ<br>system that's targeted<br>by etformin	Molecular mechanism of action	Physiological response and ARD outcome
1. Heart	AMPK activation leads to: reduction in NFkB, inhibition<br>of the JNK pathway, inhibition of AT1R,<br>upregulation of SIRT3, and GLUT4 translocation,<br>etc.	Lowered inflammation in the heart muscle,<br>decreased ROS production and endoplasmic reticulum<br>stress in cardiomyocytes, reduced cardiomyocytes<br>hypertrophy, mitochondrial protection and<br>metabolism balancing effect in cardiomyocytes<br>(thanks to SIRT3 act. and GLUT4 translocation).<br>Protective effect against cardiomyopathy and heart<br>failure.
2. Liver	Mitochondrial respiratory complex I inhibition,<br>AMPK activation. Decreased ROS production in<br>hepatocytes.	Reduced gluconeogenesis,<br>reduced glycogenolysis, and reduced fatty acid oxidation in the hepatocytes.<br>Protective against NAFLD and I/R liver injury.
3. The skeletal system	Inhibition of the PPAR- and activation of Runx2	Increased bone formation and inhibited adipogenesis<br>in skeletal tissue cells. Possibly protective<br>against osteoporosis. Potentially osteogenic.
4. Brain	Activation of the AMPK and CRRB pathway. The<br>glucose metabolism stabilising effect. Decreased<br>ROS production in neuronal mitochondria.	BDNF expression upregulation, leading to higher<br>BDNF levels, reduced oxidative stress in the brain.<br>Protective against increased dementia risk.

A possible setback in researching metformin as a life-extension and healthspan improvement agent might be the apparent lack of pharmacogenetic research and the failure to control for metabolic variations among individuals enrolled in human studies. It is believed that metformin acts »selectively,« and that various experiments related to obesity, cancer, and lifespan vary depending on sex, age, and other factors. Further research is needed to examine the direct and indirect effects of glucose intolerance, tissue damage, and the IGF-1 system, which can be assessed using pharmacogenetic factors. These may also be crucial for the ageing process, especially when evaluating pathways associated with mTOR. It is for this reason that, before metformin becomes a mainstay anti-ageing treatment, a more granular understanding of the drug's effects in humans and their dependence on genetic polymorphism is warranted [83] [84] [85].

## Conclusion

Based on extensive data and scientific research related to metformin, we can conclude that, in addition to being used as a medication for diabetes (T2DM), it also shows positive effects regarding ageing and longevity. Many studies have addressed this issue and confirmed its positive impact on ageing in various organism models. From these numerous studies, we can conclude that its effects depend on concentration, as some studies found higher concentrations to be toxic and did not contribute to life extension. We have seen that metformin can act independently, but it also shows benefits when combined with other drugs, such as rapamycin.

In addition to its effects on the ageing process and life extension, as well as numerous diseases related to ageing, metformin also effectively protects against many cardiovascular diseases linked to diabetes or its consequences. Moreover, it has antitumour effects, reducing tumour size, with its impact depending on the concentration and tumour location. Some respiratory system protective effects are evident in the literature, along with neuroprotective effects and positive effects in reducing osteoporosis and sarcopenia. All of these findings imply that metformin, more than a debatable life-extending drug, is undoubtedly a good healthspan-improving drug, at least in the T2DM patients and those with neurodegenerative and heart conditions. Such healthspanimproving effects seem to be closely related to its metabolism-stabilising effects, particularly in the mitochondria. Overall, many proposed life-extending and healthspan-improving molecular mechanisms share numerous signal pathway components with those responsible for metformin's metabolic effects.

Metformin is a drug whose positive effects are not yet fully understood. What is needed now are additional studies and testing on patients with diabetes, as well as on those who have not been diagnosed with T2DM, to compare the effects of metformin and consolidate its mechanisms of regulation.

Additionally, metabolic profiling of study participants would be highly valuable because of their genetic and metabolic polymorphisms. This is why some tests may produce an overall false negative result, even when the dosage has been adjusted to best address these polymorphism-related differences. Metformin appears to act »selectively,« and various experiments related to obesity, cancer, and lifespan show variable results depending on sex, age, and other factors. Further research is necessary to explore the direct and indirect effects of glucose intolerance, tissue damage, and the IGF-1 system, which can be evaluated using pharmacogenetic factors. Accurate personalised dosing, calculation protocols, and methods appear to be the future of metformin's anti-ageing and healthspan-enhancing research. This approach aims to avoid the potential adverse outcomes of overly inhibiting key pathways such as IGF and IGF1, which could paradoxically lead to opposite effects. Lastly, synergistic effects with other popular anti-ageing drug candidates (such as berberine, rapamycin, etc.) remain an intriguing and promising avenue for this type of research.

## Dodatak

### List of abbreviations

ARD, age/ageing-related disease;<br>BA, biological age;<br>CAR, chimeric antigen receptor;<br>CRTC-1/CREB, CREB-regulated transcription coactivator 1;<br>GDP guanosine diphosphate;<br>I/R, ischemia/reperfusion;<br>LGSI, low-grade systemic inflammation;<br>MRFTA, mitochondrial free radical theory of ageing;<br>mtROS, mitochondrial reactive oxygen species;<br>NAFLD, non-alcoholic fatty liver disease;<br>NAMPT, nicotinamide phosphoribosyltransferase;<br>NLR, NOD-like receptors;<br>RON, reactive nitrogen species;<br>Runx2, Runt-related transcription factor 2.

### AI statement

The author did not use artificial intelligence to compile this thesis.

### Funding

The authors received no funding for this work.

### Ethics approval and consent to participate

Not applicable.

### CRediT authorship contribution statement

Slavica Vujovic: Visualisation, Validation, Software, Formal analysis, Investigation, Writing-original draft. Svetlana Perovic: Methodology, Conceptualisation, Investigation. Milorad Vlaovic: Supervision, Software, Writing-review and editing. Stasa Scepanovic; Investigation, Formal analysis. Aleksandra Filipovic: Investigation, Resources.

### Conflict of interest statement

All the authors declare that they have no conflict of interest in this work.
